# Modulating Wine Aromatic Amino Acid Catabolites by Using *Torulaspora delbrueckii* in Sequentially Inoculated Fermentations or *Saccharomyces cerevisiae* Alone

**DOI:** 10.3390/microorganisms8091349

**Published:** 2020-09-04

**Authors:** M. Antonia Álvarez-Fernández, Ilaria Carafa, Urska Vrhovsek, Panagiotis Arapitsas

**Affiliations:** 1 Departamento de Nutrición y Bromatología, Toxicología y Medicina Legal, Facultad de Farmacia, Universidad de Sevilla, 41012 Sevilla, Spain; farmamari@gmail.com; 2 Department of Food Quality and Nutrition, Research and Innovation Centre, Fondazione Edmund Mach, 38010 San Michele all’Adige, Italy; ilaria.carafa@fmach.it (I.C.); urska.vrhovsek@fmach.it (U.V.)

**Keywords:** nitrogen metabolism, chardonnay, pinot gris, sulfonation, wine atypical aging, tyrosol, bitter taste, non-*Saccharomyces* yeast, winemaking

## Abstract

Yeasts are the key microorganisms that transform grape juice into wine, and nitrogen is an essential nutrient able to affect yeast cell growth, fermentation kinetics and wine quality. In this work, we focused on the intra- and extracellular metabolomic changes of three aromatic amino acids (tryptophan, tyrosine, and phenylalanine) during alcoholic fermentation of two grape musts by two *Saccharomyces cerevisiae* strains and the sequential inoculation of *Torulaspora delbrueckii* with *Saccharomyces cerevisiae*. An UPLC-MS/MS method was used to monitor 33 metabolites, and 26 of them were detected in the extracellular samples and 8 were detected in the intracellular ones. The results indicate that the most intensive metabolomic changes occurred during the logarithm cellular growth phase and that pure *S. cerevisiae* fermentations produced higher amounts of N-acetyl derivatives of tryptophan and tyrosine and the off-odour molecule 2-aminoacetophenone. The sequentially inoculated fermentations showed a slower evolution and a higher production of metabolites linked to the well-known plant hormone indole acetic acid (auxin). Finally, the production of sulfonated tryptophol during must fermentation was confirmed, which also may explain the bitter taste of wines produced by *Torulaspora delbrueckii* co-fermentations, while sulfonated indole carboxylic acid was detected for the first time in such an experimental design.

## 1. Introduction

Wine is an ancient beverage obtained mainly by the fermentation process of grape juice or must (juice and solids), which results in partial or complete transformation of the sugars principally into ethanol and CO_2_. During alcoholic fermentation, the action of yeasts produces a large number of metabolites, and the combination of yeast strain(s) and must(s) is the major key to determine the quality of the final product, as different metabolite proportions make each wine unique. In winemaking, *Saccharomyces cerevisiae* strains dominate over non-*Saccharomyces*, which are also present in the must at the beginning of fermentation. For a long time, the presence of non-*Saccharomyces* strains, which are normally detected in grapes, was considered negatively in terms of the final characteristics of wine because they produce high concentrations of acetic acid and off-flavour and because some of them are even considered spoilage microorganisms [1]. Nevertheless, nowadays, many studies confirm that the influence of non-*Saccharomyces* on wine characteristics is not always negative [2,3,4,5,6,7,8]. Lately, some studies proved that some non-*Saccharomyces* strains are able to improve the acidity, aromatic complexity, glycerol content, ethanol reduction, mannoproteins, anthocyanins and polysaccharide concentrations and can even produce bioactive compounds [8,9,10,11]. The growing interest in improving the chemical composition and sensory properties of wine has made the demand for new yeast strains that are adapted to different types of wine elaborations to grow. *Torulaspora delbrueckii* is one of the most studied and used non-*Saccharomyces* yeast because of its positive contribution to the wine sensorial characters [8]. In fact, in the market, at least 12 commercial starter cultures exist from eight companies, containing *Torulaspora delbrueckii* [8]. However, most of the non-*Saccharomyces* strains show limited fermentation capabilities, like low fermentation power and poor SO_2_ resistance [12]. Therefore, co-fermentation and sequential inoculation strategies between non-*Saccharomyces* and *S. cerevisiae* strains have gradually been adopted [2,3,13].

**Figure 1:**
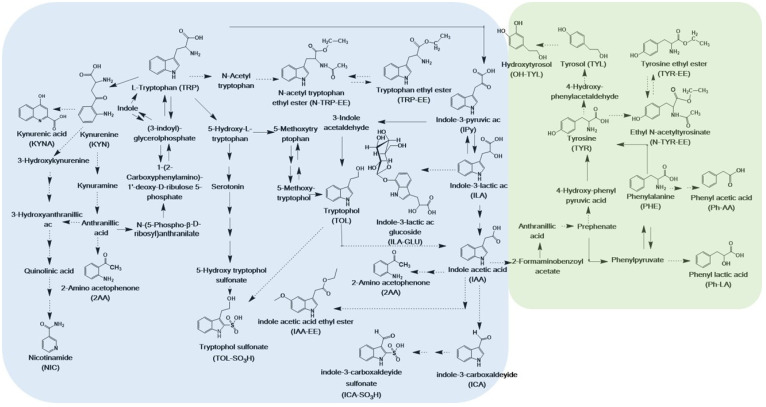
Scheme of the proposed pathway of aromatic amino acids metabolism: The blue box (**left**) includes the metabolites related to the tryptophan metabolism studied in this work. The green box (**right**) includes the metabolites related to the tyrosine and phenylalanine metabolisms. Possible biosynthetic pathways leading to the de novo synthesis of three aromatic amino acids are included. The metabolites that include abbreviation in parentheses were measured by the UPLC-MS/MS method.

Organic and inorganic nitrogen availability is a key parameter for grape must fermentation, able to influence wine quality and composition. Nitrogen affects yeasts growth, fermentation kinetics and flavour production and is commonly added to the must for boosting the growth of yeasts in suboptimal concentrations [14]. The sources of nitrogen in fermentation include inorganic nitrogen and amino acids, which are common ingredients in must. Regarding *S. cerevisiae,* it is known that the preferential sources of nitrogen are ammonia, arginine, aspartic acid, leucine, isoleucine, lysine, methionine, serine, threonine, and asparagine. The aromatic amino acids phenylalanine (PHE), tyrosine (TYR) and tryptophan (TRP), which take part in the grape metabolome, can also be consumed but are less preferable [15]. On the other hand, the catabolites of these aromatic amino acids include several sensorial and biological active compounds, such as tryptophol (TOL), tyrosol (TYL), hydroxytyrosol (OH-TYL) kynurenic acid (KYNA), kynurenine (KYN), indole acetic acid (IAA), indole lactic acid (ILA), and ethyl esters of TRP (TRP-EE) and TYR (TYR-EE) (Figure 1) [9,16]. 

Via the Ehrlich pathway, the amino acids produce aromatic higher alcohols, like TOL and TYL, for which biosynthesis is positively correlated with ethanol stress-tolerant yeast, and as auto inducers are able to transmit information about the population density and the amount of available nitrogen [17]. These higher alcohols at high concentrations can result in a strong, pungent smell and taste, whereas optimal concentrations have positive impacts on producing wines with flowery character [17,18]. In addition, the TRP yeast metabolism can contribute to wine aroma directly by bio-transforming odourless metabolites into flavour-active compounds, like methyl mercaptan and indole [17], and indirectly through chemical reactions in wine producing aromatic substances like 2-aminoacetophenone (2AA) [19] (Figure 1).

Gougeon et al. [20], by applying a Fourier-transform ion cyclotron resonance mass spectrometry based untargeted analysis, showed that the metabolites of aromatic amino acid metabolism (PHE, TYR and TRP) characterized more white wine metabolomes, while the methionine, alanine, aspartate, glycine, serine, threonine, arginine and proline metabolomic pathways characterized more red wine metabolomes. Lately, the three amino acids and the metabolites related to TRP catabolism were demonstrated to be helpful in the discrimination of monovarietal wines [21]. The behaviour of several of these aromatic amino acid catabolites is still not well known (if not unknown) under real grape must fermentation conditions. Moreover, although nitrogen availability is crucial in winemaking, the kinetics of the aromatic amino acids and their metabolites in co-fermentation conditions between non-*Saccharomyces* and *S. cerevisiae* are not well studied yet.

A recent work of our group focused on the intra- and extracellular metabolic dynamics of the three aromatic amino acid catabolites on synthetic media during fermentation of two *S. cerevisiae* and one non-*Saccharomyces* strains [9]. The study demonstrated that all three strains were able to produce 2AA and sulfonated tryptophol (TOL-SO_3_H), two previously considered metabolites formed only during wine aging and storage. The presence of 2AA in wine is correlated with the off-flavour of atypical aging, while recent investigations indicate that TOL-SO_3_H contributes negatively to white wine by adding bitterness [22]. In addition, the above study showed that *Torulaspora delbrueckii* produced minor amounts of 2AA and higher amounts of KYN and KYNA as compared to the *S. cerevisiae* strains.

The aim of the present project was to reproduce the previous work in two must batches in order to verify if the same results are reproducible in real grape juice fermentations. A high throughput-targeted UPLC-MS/MS method was used to monitor the metabolites of the aromatic amino acid from both intra- and extracellular metabolism during alcoholic fermentation of two grape musts driven by *two S. cerevisiae* and one *Torulaspora delbrueckii* yeast strains. As an additional aim, we studied the kinetics of the same metabolites in case of sequential fermentation (started by *T. delbrueckii* and continued by *S. cerevisiae*).

## 2. Materials and Methods 

### 2.1. Reagents and Materials

All chemicals used in this study were of the highest purity grade available and were purchased from Sigma-Aldrich (Madrid, Spain or Milan, Italy), Chengdu Biopurify Phytochemicals Ltd. (Chengdu, Sichuan, China) unless otherwise stated (Table S1). Tryptophol sulfonated was prepared as previously described [23].

### 2.2. Yeast Strains

Three commercial wine yeast strains were used in this study: *S. cerevisiae* Lalvin YSEO QA23^®^ (Lallemand) (QA), *S. cerevisiae* Red Fruit RF^®^ (Enartis) (RF) and *T. delbrueckii* TD291 Biodiva™ (Lallemand) (Td).

### 2.3. Alcoholic Fermentation

The alcoholic fermentations were performed in two grape musts from chardonnay and pinot gris white grapes (vintage 2017), sourced from the Fondazione Edmund Mach experimental winery. The fermentation processes were carried out by QA, RF, and Td commercial yeast strains. Each fermentation included four biological replicates and one control, for a total of 8 fermented and 2 control samples. The must was aliquoted into bottles and pasteurized (80 °C for approximately 5 min) in order to eliminate the autochthonous microflora. Sterile (NH_4_)_2_SO_4_ (0.3 g/L in chardonnay and 0.1 g/L in pinot gris) and thiamine (0.4 mg/L) were added to both musts. Each batch of must (450 mL) was inoculated with 5 × 10^6^ colony forming units (CFU)/mL of QA, RF or Td; capped with taps equipped with a capillary to release carbon dioxide; and incubated at 18 °C in static conditions as for wine making until the fermentations were considered finished (no more weight loss was quantified [24]). During the experiment, which lasted 20 days, a total of 9 or 10 samples were collected from each fermentation or control bottle in a microbiological laminar hood. To monitor the fermentation, the bottles were weighed before and after sampling. Cellular growth evolution was monitored daily by measuring the optical density (OD_600nm_) and plate count on the Yeast Potato Dextrose (YPD) agar and Plate Count Agar (PCA) (Oxoid LTD, Basngstoke, Hampshire, UK). PCA plates were incubated for 24 h at 30 °C, and YPD plates were incubated for 72 h at 25 °C. After 4 or 5 days of fermentation, the four batches of *T. delbrueckii* of both musts were subjected to sequential inoculation with *S. cerevisiae* QA23 (5 × 10^6^ CFU/mL). This kind of fermentation was performed to simulate wine making with non-*Saccharomyces* strains, which are unable to survive when high concentrations of ethanol are present and cannot finish the alcoholic fermentation [2].

### 2.4. Extracellular and Intracellular Metabolite Extraction

The sampling frequency for each batch is showed in Tables S2–S5. Samples were centrifuged at 4 °C at 13,000× *g* for 15 min in order to separate the cells from the supernatant. The supernatant, namely the extracellular samples, was filtered with a 0.2-µm filter in order to remove any residual cell, was finally aliquoted and was stored at −20 °C until further analysis, whereas cells were washed twice with PBS and subjected to quenching and intracellular extraction as previously reported [11] (based on a Villas-Boas et al. method [25]), with some modifications related to the centrifugation temperature, which was set up at 4 °C. Indeed, according with a previous study [11], no differences were found between centrifugation at −20 °C and 4 °C in the cold cell extraction procedure. The extracts obtained were stored at −80 °C until analysis.

The intracellular extracts were cleaned up by filtration at 30 mg/well in Phree 96-well plates as previously reported by Alvarez-Fernandez et al. [11] in order to remove the phospholipids and proteins that could interfere in chemical analysis. A scheme of the extraction procedure is shown in Figure S1.

### 2.5. UPLC-MS/MS Instrumental Analysis

The analyses were performed according to Arapitsas et al. [23]. Briefly, the separation of the metabolites was achieved on a Waters Acquity UPLC system equipped with an HSS T3 column 1.8 μm, 150 mm × 2.1 mm (Milford, MA, USA). The injection volume of both standard solutions and samples was 10 μL, and the samples were kept at 6 °C during analysis. Mass spectrometry detection was performed on a Waters Xevo TQ-MS (Milford, MA, USA) instrument equipped with an electrospray (ESI) source. Data processing was done using Waters TargetLynx tools of the MassLynx 4.1 software. In order to control the robustness of the system and the stability of the signal, tryptophan deuterated d5 was used as an internal standard (1.33 ppm) and the order of the sample injections was randomized, and to control the instrumental variability, the QC samples were injected every 10 sample injections. The QC samples were prepared as a pooled mix of extracellular and intracellular samples separately.

### 2.6. Statistical Analysis and Data Visualization

ANOVA and the post hoc Tukey’s HSD (honestly significant difference) tests were performed with the Statistica software, version sixteen. *p* values < 0.05 were associated with statistical significance between groups (Tables S2–S5). The software EZInfo SIMCA-P, version 12.0.0 (Umetrics, Umea, Sweden) was used to create the principal component analysis biplot (PCA-biplot). MetaboAnalyst was used to create heatmaps [26]. The software SPSS 19.0 (IMB software) was used for the kinetics plots. The software Graphad Prism 6 for windows, version 6.01 was used for the cell growth plots.

## 3. Results and Discussion

**Figure 2:**
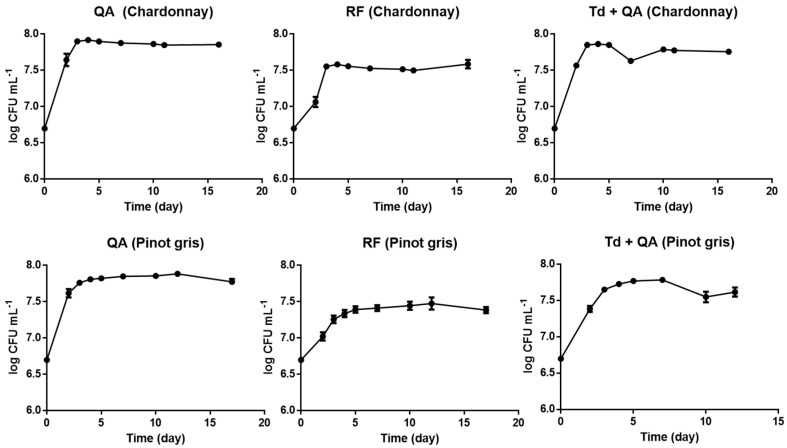
Growth dynamics of yeasts in the two musts during the fermentation. QA: *S. cerevisiae* QA23; RF: *S. cerevisiae* RF; Td: *T. delbrueckii*; and TQ: *T. delbrueckii + S. cerevisiae* QA.

The evolution of cellular growth, as shown in Figure 2, was typical for both *S. cerevisiae* pure fermentations and *T. delbrueckii + S. cerevisiae* sequential fermentations during winemaking [16,27]. Generally, in all cases, the amount of cell cultures increased rapidly during the initial 2–3 days from approximately 10^6^ CFU/mL to 10^7–8^ CFU/mL and then remained stable until the end of the experiment. The presence of bacteria was not detected at any time point on PCA plates, suggesting that bacteria did not interfere during the fermentation process.

### 3.1. Extracellular Metabolites 

The majority of published works about non-*Saccharomyces* use in winemaking focuses on the volatile metabolites and the basic oenological parameters [8,9,10,11,28]. Only a few papers deal with measurement of the metabolites produced by the aromatic amino acids [9,11,29,30], even though they include several important sensorial and biological active compounds (see the Introduction section). This project tried to enrich our knowledge with regard to a big part of the metabolites presented in Figure 1.

**Figure 3:**
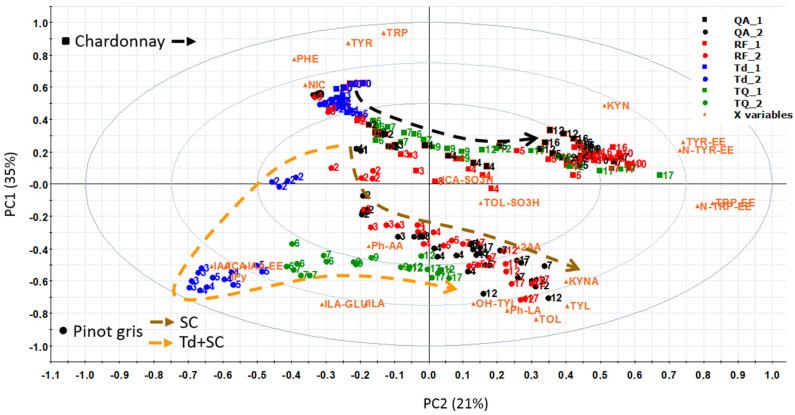
Principal component analysis biplot (PCA-biplot), including both samples and loadings (metabolites), based on the measured extracellular metabolites: QA_1, RF_1, Td_1 and TQ_1 refer to chardonnay must (upper part); QA_2, RF_2, Td_2 and TQ_2 refer to pinot gris must (low part). The numbers indicate the days of fermentation, and the red letters indicate the metabolites. The arrows indicate the metabolomic profile evolution during fermentation. QA: *S. cerevisiae* QA23; RF: *S. cerevisiae* RF; Td: *T. delbrueckii*; and TQ: *T. delbrueckii + S. cerevisiae* QA.

The number of identified and quantified metabolites was higher in extracellular samples than in intracellular ones, which is in accordance with the results of our previous study [9]. The UPLC-MS/MS method used was able to quantify 33 compounds in wine [23], 26 of which were detected and quantified in the extracellular and 8 were detected in the intracellular samples in this experimental design. The PCA-biplot in Figure 3 was based on the results of the extracellular metabolites and includes the fermentation of both musts. PC1 separates the two musts, with chardonnay samples on the top of the graph and pinot gris samples on the bottom. On the left side are the samples of the first days of fermentation, and as we move to the right, we find samples from the end of fermentation. In the case of pinot gris, we noticed that, initially, the Td fermentations were different from the one obtained with the pure *S. cerevisiae* samples, but after inoculation with *S. cerevisiae*, the sequential inoculation samples moved closer to the pure *S. cerevisiae* fermentation samples, and at the end of fermentation, they clustered together.

**Figure 4:**
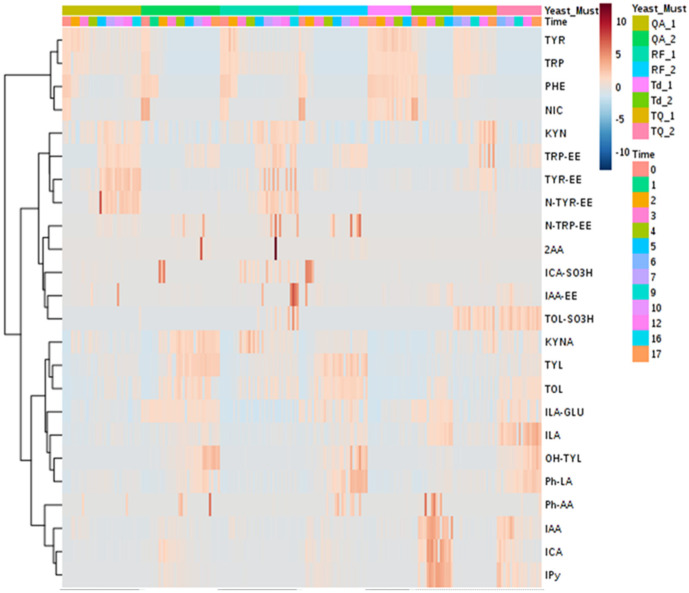
Clustered heatmap based on the measured extracellular metabolites: QA_1, RF_1, Td_1 and TQ_1 refer to the chardonnay must, and QA_2, RF_2, Td_2 and TQ_2 refer to the pinot gris must. The Time indicates the fermentation day. QA: *S. cerevisiae* QA23; RF: *S. cerevisiae* RF; Td: *T. delbrueckii*; and TQ: *T. delbrueckii + S. cerevisiae* QA23.

**Figure 5:**
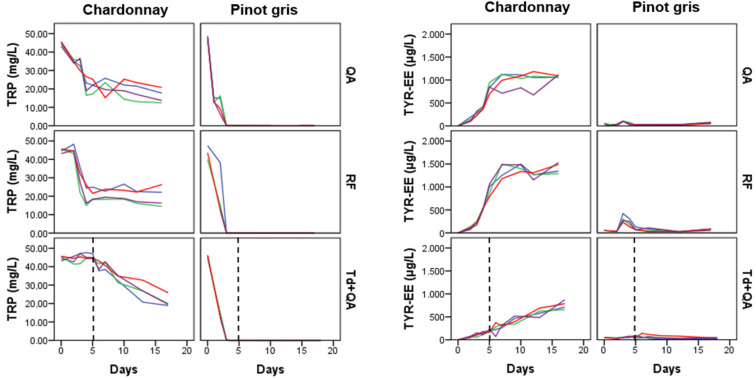
Kinetics of Tryptophan (TRP) and Tyrosine ethyl ester (TYR-EE) in the extracellular samples of the two musts: The vertical black dashed line separates the Td fermentation alone (**left**) from the co-inoculated fermentations of Td+QA (**right**). Each coloured line corresponds to one of the four replicated fermentations.QA: *S. cerevisiae* QA23; RF: *S. cerevisiae* RF; and Td: *T. delbrueckii*.

The heatmap in Figure 4 is an alternative way to visualize the same PCA-biplot results of Figure 3. Both graphs show that the three aromatic amino acids (TRP, PHE, and TYR) and nicotinamide (NIC) clustered in the same group. These metabolites were detected at high concentrations at the beginning of fermentation and then they decreased in both musts. Even though both musts had a similar initial concentration of TRP, PHE and TYR, in pinot gris, they were consumed much faster, probably due to the minor content of inorganic nitrogen. After 4–5 days of fermentation, the amino acids were present in traces in pinot gris (about 0.1–0.2 mg/L), whereas chardonnay contained about 7 mg/L of TYR, 0.3 mg/L of PHE and 21 mg/L of TRP (Tables S2 and S3). In addition, in QA and RF fermentations, the amino acids were consumed much faster than in samples sequentially inoculated with *T. delbrueckii* and *S. cerevisiae* QA23, with a trend similar as the one of TRP in Figure 5 (see also Table S2 and Figures S2–S4). The preference of Td for ammonium sulphate and then for amino acids as nitrogen sources [31] might explain this data. These results could be a positive indication that *T. delbrueckii* has different orders of nitrogen preference compared to *S. cerevisiae*, and so its impact on sequentially fermentation is not so competitive as far as nitrogen consumption is concerned. Therefore, using *T. delbrueckii* as a starting culture in winemaking should not influence the growth and the metabolic activity of *S. cerevisiae* (Figure 2) too badly. Generally, the results about the three amino acids’ consumption during fermentation are in accordance with the literature [9,15,16,24,31], since their concentration decreased quickly mainly during the logarithmic phase of cell growth (Figure 2) by covering the needs of the yeasts in nitrogen. Finally, chardonnay must fermented by *S. cerevisiae* QA and RF showed an increase of PHE and NIC concentrations in the last part of fermentation (Table S2 and Figures S3 and S4). A similar evolution was registered also in our previous work on synthetic must [9] and was explained as a possible *de novo* synthesis. More specifically, PHE in chardonnay had a mean concentration of 15.4 mg/L at the beginning of fermentation; after 5 days, it decreased to 0.3 mg/L, and at the end of the experiment (16 days), it was 7.9 mg/L (Table S2 and Figure S3). According to Braus [32] and Toyn et al. [33], chorismic acid and anthranilic acid (both not measured in this work) can be two intermediates of the aromatic amino acids biosynthesis in *Saccharomyces cerevisiae* (Figure 1).

A second group of metabolites (Figure 4) included the amino acid ethyl esters TRP-EE, TYR-EE and N-TYR-EE and the amino acid-like KYN (Figure 1). In both musts, generally these metabolites increased during fermentation. Especially for the chardonnay *S. cerevisiae* fermentations, we noticed that the concentration of this group of metabolites followed cell growth behaviour, with a fast increase during the logarithmic phase of cell growth and a relatively stable concentration during the cell stationary phase, with TYR-EE behaviour being a typical example (Figure 5). On the other hand, the concentration of these metabolites in chardonnay must sequentially inoculated with *T. delbrueckii* and *S. cerevisiae* QA23 continued to rise constantly during the whole experiment (Figure 5, Table S2 and Figures S6 and S7).

Then, we had a third group of catabolites (Figure 4) of the three aromatic amino acids, which included indolic metabolites (ILA and KYNA), fusel alcohols (TYL, TOL and OH-TYL) and Ph-LA (Figure 1). These metabolites characterized the metabolism of all yeasts, and they all increased during fermentation. TYL, TOL, KYNA and ILA increased the most during the first five days of fermentation (Tables S2 and S3 and Figures S9–S14), which corresponded to the logarithmic phase and the beginning of the stationary phase of yeast growth. OH-TYL presented a constant increase during the whole fermentation period. Again, these metabolites presented their maximum mean concentration in pinot gris, with TYL quantified at up to 22.4 mg/L (max for chardonnay was 7.8 mg/L), OH-TYL at 23.3 mg/L (max for chardonnay was 26.8 µg/L), TOL at 50.0 mg/L (max for chardonnay was 29.1 mg/L), KYNA at 0.8 mg/L (max for chardonnay was 0.9 mg/L), ILA at 324 µg/L (max for chardonnay was 94 µg/L) and Ph-LA at 6.0 mg/L (max for chardonnay was 1.4 mg/L). Moreover, pinot gris must sequentially inoculated fermentations presented statistically significant lower maximum concentration for TYL and TOL than for fermentation where only the *S. cerevisiae* strain was used. The biosynthesis of TYL and TOL is related to nitrogen depletion, since in nitrogen-limiting conditions, yeast can catabolize assimilated amino acids (TYR and TOL, respectively) through the Ehrlich pathway to obtain the needed nitrogen (Figure 1) [34]. These higher alcohols, quorum sensing molecules, are able to transmit information about the population density and the amount of available nitrogen, while excessive amounts of TYL and TOL can result in a strong pungent smell and taste [34]. OH-TYL is a potent antioxidant with a molecular structure similar to TYL, found mainly in olive oil. It is possible that production of OH-TYL proceeds from TYL, but in practice, the relevant hydrolase was not isolated in yeast but in bacteria [35]. OH-TYL was detected in wine, and recently, it was demonstrated that it can be produced by yeast [10,36]. Since the highest concentration quantified in pinot gris must was about 90 times higher than that of chardonnay while the maximum differences in the three fermentations (QA, RF and Td+QA) were less than 20%, it can be hypothesized that OH-TYL production depends mainly on must/grape cultivar composition. The concentrations and the kinetics of production for TYL, OH-TYL, Ph-LA, ILA and KYNA were similar among the four replicates. However, the formation of TOL was irregular, especially in chardonnay (Tables S2 and S3 and Figures S9–S14).

**Figure 6:**
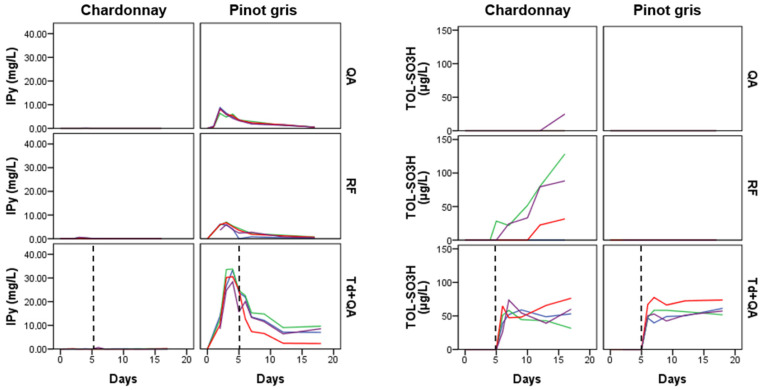
Kinetics of Indole pyruvic acid (IPy) and Tryptophol sulfonate (TOL-SO_3_H) in the extracellular samples of the two musts: The vertical black dashed line separates the Td fermentation alone (**left**) from the co-inoculated fermentations of Td+QA (**right**). Each coloured line corresponds to one of the four replicated fermentations. QA: *S. cerevisiae* QA23; RF: *S. cerevisiae* RF; Td: *T. delbrueckii*; and TQ: *T. delbrueckii + S. cerevisiae* QA.

Indole acetic acid (IAA), indole carboxaldehyde (ICA) and indole pyruvic acid (IPy) clustered together and, as shown in the heatmap in Figure 4, characterized the sequentially inoculated fermentations (Td+QA). The three metabolites are biosynthetically connected, since starting from TRP, we obtain first the biosynthesis of IPy, then IAA and finally ICA (Figure 1). Their concentration increased substantially within the first days of yeast fermentation and then decreased again, forming a peak during the logarithmic phase of the cell growth (Figure 6, Figures S15 and S16). The peak with the highest concentration was registered on the 3rd–4th day. The maximum mean values were detected for Td fermentations in pinot gris, with 2.6 mg/L ICA (1.1 mg/L for QA and 0.7 mg/L for RF), 1.7 mg/L IAA (0.4 mg/L for QA and 0.5 mg/L for RF) and 31.5 mg/L IPy (7.9 mg/L for QA and 6.3 mg/L for RF) (Tables S2–S3). This finding confirmed the results of our previous work on synthetic must, where the abovementioned metabolites had the same trend and made a cluster [9]. The auxin IAA is best known for its role in plant cell elongation, division and differentiation, but recent studies suggest that yeasts are also able to synthesize it [30,37]. The role of IAA in yeasts is still not clear; however, the experiment by Prusty Rao et al. [37] suggested that yeast may have multiple pathways for IAA synthesis, one of which is not dependent on TRP. The data of our experiment indicate that IAA is formed during the cell growth phase, but afterward, its concentration decreased very fast, in counter to the quorum sensing molecules (TOL and TYL) for which concentration remained stable during the cell stationary phase (Figures S10, S12 and S16).

A final group of metabolites worth mentioning includes the sulfonated compounds tryptophol sulfonate (TOL-SO_3_H), indole-3-acetaldehyde sulfonate (ICA-SO_3_H) and 2-aminoacetophenone (2AA). As stated by the strain manufacturer, QA and Td produce low SO_2_ quantities (3–4 mg/L) while RF produces medium quantities (8–20 mg/L) thereof. For the chardonnay must, we quantified the maximum mean concentrations of the sulfonated TOL-SO_3_H and ICA-SO_3_H for the RF experiments, thus 62 µg/L of TOL-SO_3_H and 23.3 µg/L of ICA-SO_3_H. To the best of our knowledge, this is the first time this compound (ICA-SO_3_H) is detected and quantified in wine and during must fermentations. The maximum ICA-SO_3_H mean concentrations for the sequential inoculation *T. delbrueckii* + *S. cerevisiae* QA experiment were registered after the *S. cerevisiae* QA addition in chardonnay must and were similar to those of the *S. cerevisiae* QA experiment (about 5.2 µg/L) (Tables S2 and S3). We did not detect ICA-SO_3_H in the sequential inoculation of pinot gris. Moreover, ICA-SO_3_H appeared generally earlier than TOL-SO_3_H, probably because aldehydes preferably react with SO_2_ and react with alcohols [16] to a lesser extent. The peak of ICA-SO_3_H was registered in the middle-early stages of the fermentations, like for ICA; thus, ICA-SO_3_H was formed when ICA was available (Tables S2 and S3 and Figures S15 and S18). As far as TOL-SO_3_H is concerned (Figure 6) and in accordance with our previous work [9], we detected the highest concentration for the RF experiment, which is the strain with the highest SO_2_ production capabilities. For the QA experiment, measurable amounts of TOL-SO_3_H (one from the 72 samples analysed) were present only in one fermentation bottle (after 16 days). Even though both QA and Td are low SO_2_ producers and only Td was present in the must, no TOL-SO_3_H was detected; after the addition of QA in the sequential inoculation experiment, we noticed a big increase in its concentration (Figure 6). This phenomenon deserves further investigation to understand if sequential inoculations can cause an increase in the SO_2_ production, especially for the production of organic/biological wines. The sulfonation of tryptophol in wine was described for the first time in bottled white wines recently [38], and as reported by Alvarez-Fernandez et al. [9], the reaction can occur in anaerobic fermentation too. A hypothesis we cannot discount is that the sulfonation of ICA and TOL is used by yeast to control the activity of these two compounds or their biosynthetic pathway. Lately, TOL-SO_3_H was reported as a potential contributor to bitterness in white wines, which is an unacceptable taste commonly attributed to flavanols [22]. Two independent works, one focused on Barbera red wine and the second on sauvignon blanc white wine, reported that sequentially inoculated fermentations of Td+QA produced wines characterized by a bitter attribute [6,7]. Whether TOL-SO_3_H is responsible for the bitterness of such a winemaking protocol is something that requires further validation. 2AA, which is an off-odour compound responsible for the atypical aging wine defect, could be produced by oxidative degradation of IAA and KYN or enzymatically from anthranilic acid [19]. 2AA was quantified in small amounts (0.1–0.5 μg/L) and in few samples, only in musts fermented by *S. cerevisiae* RF and QA, and was not detected in any of the sequential inoculation samples. This result is in accordance with our previous experience with synthetic must [9], proving that sequential inoculation fermentations produce lower amounts of this metabolite.

Finally, it was interesting to notice that the N-acetyl ethyl esters of tryptophan and tyrosine (N-TRP-EE and N-TYR-EE) and their corresponding ethyl ester (TRP-EE and TYR-EE) were produced in much higher concentrations in the *S. cerevisiae* fermentations. Since the same issue occurred also in our previous work with synthetic must, it could be worth our while to explore whether this biosynthetic route is characteristic only of *S. cerevisiae* stains. N-TYR-EE participates in the regulation of TRP synthesis and metabolism in yeasts, as a tryptophan synthase inhibitor [39,40] and by mediating the production of TOL [41]. Moreover, deacetylation is a common mechanism of metabolite deactivation and/or detoxification [42,43]. Additionally, a recent study that quantified these metabolites in about 200 wines (including several chardonnay and pinot gris) demonstrated that red wines have much higher concentrations of N-TRP-EE and TRP-EE than white wines, even if they had generally similar TRP amounts [23]. All these are indications that the above metabolites play a strategic role in the yeast mechanism, machinery and regulation and thus in fermented food quality.

### 3.2. Intracellular Metabolites

**Figure 7:**
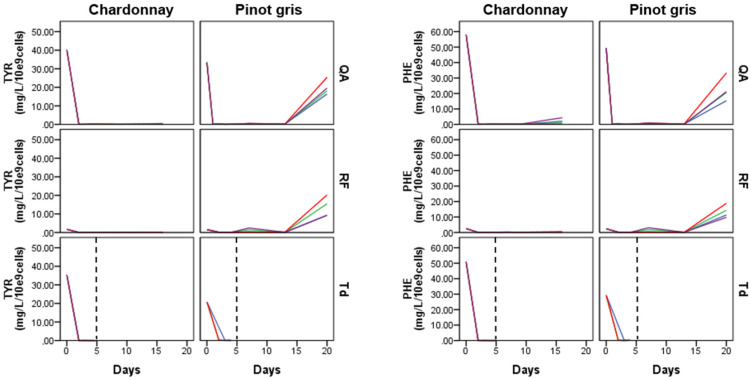
Kinetics of tyrosine (TYR) and phenylalanine (PHE) in the two musts intracellular samples: The vertical black dashed line indicates the QA addition time. Each coloured line corresponds to one of the four replicated fermentations. QA: *S. cerevisiae* QA23; RF: *S. cerevisiae* RF; and Td: *T. delbrueckii*.

The intracellular samples of both musts were characterized by the predominance of the three aromatic amino acids (TYR, PHE and TRP), NIC and TOL. The fact that intracellular samples were poorer in number of metabolites was also in accordance with our previous work with synthetic must [30] and other studies [44], but there might be several causes for it, such as the extraction methods and chemical characteristic of metabolites studied. Here, we noticed an increase in the concentration of the three amino acids—like a peak—in the middle (approximately 7th day) of the RF fermentation in chardonnay must (Figure 7, Figures S20–S22 and Table S5). This could be the result of the activity of amino acid transporter permease, which facilitated the entrance of the amino acids inside the cell. As already mentioned, these three amino acids are not used as a preferred nitrogen source [15] in the early phase of fermentation because other amino acids, such as lysine, are initially used by the yeasts. As a consequence, the aromatic amino acids are likely transported to the intracellular space later. In the last phases of fermentation, we also noticed an increase in the concentration of the three amino acids, which is in accordance with the extracellular data (Figures S2 and S3). Probably, this increase in aromatic amino acids in the extracellular space was caused by a de novo intracellular biosynthesis [32,33,45]. However, we registered a higher concentration of TYR and PHE in intracellular samples of pinot gris than in chardonnay must (Figure 7 and Tables S4 and S5). Probably, the presence of a low concentration of inorganic nitrogen sources stimulated the entrance of permeation of amino acids through permeases to supply cells with their nitrogen requirements [46]. TOL had a very similar trend to the behaviour of the three amino acids, with a peak in the middle (approximately 7th day) of fermentation and then a maximum value at the end (Figure S24 and Tables S4 and S5). Finally, low concentrations of KYN, KYNA and NIC were quantified in the intracellular samples (Figures S20–S22).

## 4. Conclusions

In conclusion, this work confirmed that the off-flavour molecule 2AA is formed during the fermentation process by *S. cerevisiae* yeasts while sequential inoculations of *T. delbrueckii* with *S. cerevisiae* produced lower amounts. Moreover, we confirmed that the sulfonation of tryptophol can occur during fermentation, and we detected ICA-SO_3_H in fermented must for the first time. Even if both *S. cerevisiae* QA and *T. delbrueckii* are low SO_2_ producers and separately did not originate the bitter taste TOL-SO_3_H, when they were both present in the must, we registered a high production of such a taste. This particularity could maybe explain the bitter taste found in wines produced by sequential inoculations of *T. delbrueckii* with *S. cerevisiae*. The N-acetyl ethyl esters of TRP and TYR were produced especially at the beginning of fermentation, during the logarithmic cellular growth phase. The data also confirmed an increase in amino acids at the end of fermentation, both in intra- and extracellular samples. We registered high concentrations of the biologically active TYL and OH-TYL, which were very stable, whereas TOL showed a higher variability, suggesting its participation in several processed, such as the reaction with SO_2_. The concentration of ILA and ILA-GLU did not decrease, indicating that they are probably not a yeast preferential nitrogen source. Finally, although sequentially inoculated fermentations showed a higher production of metabolites linked to the well-known plant hormone indole acetic acid, their metabolomic profile at the end of the fermentation linked to aromatic amino acid catabolites, was mainly influenced by the *S. cerevisiae* strain metabolism. The outputs of this work have a direct impact on winemaking protocols, shedding light on our knowledge about the metabolisms of yeasts and yeast interaction. Further investigations are necessary in order to understand yeast metabolic pathways involved in amino acid production.

## Supplementary Materials

The following are available online at https://www.mdpi.com/2076-2607/8/9/1349/s1: Figure S1. Extraction protocol. Figure S2. Kinetics of tyrosine in the extracellular samples. Figure S3. Kinetics of phenylalanine in the extracellular samples. Figure S4. Kinetics of nicotinamide in the extracellular samples. Figure S5. Kinetics of kynurenine in the extracellular samples. Figure S6. Kinetics of tryptophan ethyl ester in the extracellular samples. Figure S7. Kinetics of N-acetyl-tyrosine ethyl ester in extracellular samples. Figure S8. Kinetics of N-acetyl-tryptophan ethyl ester in the extracellular samples. Figure S9. Kinetics of kynurenic acid in the extracellular samples. Figure S10. Kinetics of tyrosol in the extracellular samples. Figure S11. Kinetics of OH-tyrosol in the extracellular samples. Figure S12. Kinetics of tryptophol in the extracellular samples. Figure S13. Kinetics of indole lactic acid in the extracellular samples. Figure S14. Kinetics of phenyl-lactic acid in the extracellular samples. Figure S15. Kinetics of indole carboxaldehyde in the extracellular samples. Figure S16. Kinetics of indole acetic acid in the extracellular samples. Figure S17. Kinetics of indole acetic acid ethyl ester in the extracellular samples. Figure S18. Kinetics of indole carbohaldehyde sulfonated in the extracellular samples. Figure S19. Kinetics of 2-aminoacetophenone in the extracellular samples. Figure S20. Kinetics of kynurenine in the intracellular samples. Figure S21. Kinetics of kynurenic acid in the intracellular samples. Figure S22. Kinetics of nicotinamide in the intracellular samples. Figure S23. Kinetics of tryptophan in the intracellular samples. Figure S24. Kinetics of tryptophol in the intracellular samples. Table S1. Standards. Table S2. Descriptive statistics of the extracellular metabolite concentrations in chardonnay and one-way ANOVA analysis. Table S3. Descriptive statistics of the extracellular metabolite concentrations in pinot gris and one-way ANOVA analysis. Table S4. Descriptive statistics of the intracellular metabolite concentrations in chardonnay and one-way ANOVA analysis. Table S5. Descriptive statistics of the intracellular metabolite concentrations in pinot gris and one-way ANOVA analysis.

## Abbreviations

2AA	2-aminoacetophenone
ABA	abscisic acid
ABA-GLU	abscisic acid glucoside
IAA	indole acetic acid
IAA-EE	indole acetic acid ethyl ester
ICA-SO_3_H	indole carboxaldehyde sulfonate
ICA	indole carboxaldehyde
ILA	3-indole lactic acid
ILA-GLU	indole lactic acid glucoside
ILA_GLU (2)	indole lactic acid glucoside isomer
IPy	indole pyruvate
KYN	kynurenine
KYNA	kynurenic acid
N-TYR-EE	N-acetyl tyrosine ethyl ester
N-TRP-EE	N-acetyl tryptophan ethyl ester
NIC	nicotinamide
OH-TYL	hydroxytyrosol
Ph-AA	phenyl acetic acid
Ph-LA	phenyl lactic acid
PHE	phenylalanine
TOL	tryptophol
TOL-SO_3_H	tryptophol sulfonate
TRP	tryptophan
TRP-EE	tryptophan ethyl ester
TYL	tyrosol
TYR	tyrosine
TYR-EE	tyrosine ethyl ester
QA	QA23 Saccharomyces cerevisiae strain of yeast
RF	RED FRUIT *Saccharomyces cerevisiae* strain of yeast
Td	*Torulaspora delbrueckii* non-Saccharomyces strain of yeast
MRM	multiple reaction monitoring
TQ	triple quadrupole
SPE	solid phase extraction
UHPLC-MS/MS	ultrahigh resolution liquid chromatography associated with mass spectrometry
RSD	standard deviation
MS	mass spectrometry analysis
YPD	yeast extract, peptone and dextrose agar medium
PCA	plate count agar
PBS	phosphate buffered saline
PCA-biplot	principal component analysis biplot
