# Supplementary material for: Modulating Wine Aromatic Amino Acid Catabolites by Using Torulaspora delbrueckii in Sequentially Inoculated Fermentations or Saccharomyces cerevisiae Alone
Source: Microorganisms. 2020 Sep 4;8(9):1349. doi: 10.3390/microorganisms8091349 (PMC7565473; doi:10.3390/microorganisms8091349)
Supplement: Supplementary file 1 [file microorganisms-08-01349-s001.zip › Suppl materials to XML conversion/Suplementary data to XML conversion.docx]

**Modulating the wine aromatic amino acids catabolites by using Torulaspora delbrueckii as a starter in sequentially inoculated fermentations**

M. Antonia Álvarez-Fernández ^1^, Ilaria Carafa ^2^, Urska Vrhovsek ^2^, Panagiotis Arapitsas ^2,*^

*^1^Departamento de Nutrición y Bromatología, Toxicología y Medicina Legal. Facultad de Farmacia. Universidad de Sevilla, 41012 Sevilla, Spain*

*^2^Department of Food Quality and Nutrition, Research and Innovation Centre, Fondazione Edmund Mach, 38010 San Michele all’Adige, Italy*

*corresponding author: panagiotis.arapitsas@fmach.it and panagiotis.arapitsas@gmail.com

**Supplementary Figures and Tables**

**Figure S1.** Scheme of the sample extraction procedure. Based on Villas-Bôas et al.

**Figure S2.** Kinetics of Tyrosine in the two musts extracellular samples (1: Chardonnay; 2: Pinot gris). The vertical black dashed line separates the Td fermentation alone (left) from the co-inoculated fermentations of Td+QA (right).

**Figure S3.** Kinetics of Phenylalanine in the two musts extracellular samples (1: Chardonnay; 2: Pinot gris). The vertical black dashed line separates the Td fermentation alone (left) from the co-inoculated fermentations of Td+QA (right).

**Figure S4.** Kinetics of Nicotinamide in the two musts extracellular samples (1: Chardonnay; 2: Pinot gris). The vertical black dashed line separates the Td fermentation alone (left) from the co-inoculated fermentations of Td+QA (right).

**Figure S5.** Kinetics of Kynurenine in the two musts extracellular samples (1: Chardonnay; 2: Pinot gris). The vertical black dashed line separates the Td fermentation alone (left) from the co-inoculated fermentations of Td+QA (right).

**Figure S6.** Kinetics of Tryptophan ethyl ester in the two musts extracellular samples (1: Chardonnay; 2: Pinot gris). The vertical black dashed line separates the Td fermentation alone (left) from the co-inoculated fermentations of Td+QA (right).

**Figure S7.** Kinetics of N-acetyl-Tyrosine ethyl ester in the two musts extracellular samples (1: Chardonnay; 2: Pinot gris). The vertical black dashed line separates the Td fermentation alone (left) from the co-inoculated fermentations of Td+QA (right).

**Figure S8.** Kinetics of N-acetyl-Tryptophan ethyl ester in the two musts extracellular samples (1: Chardonnay; 2: Pinot gris). The vertical black dashed line separates the Td fermentation alone (left) from the co-inoculated fermentations of Td+QA (right).

**Figure S9.** Kinetics of Kynureninc acid in the two musts extracellular samples (1: Chardonnay; 2: Pinot gris). The vertical black dashed line separates the Td fermentation alone (left) from the co-inoculated fermentations of Td+QA (right).

**Figure S10.** Kinetics of tyrosol in the two musts extracellular samples (1: Chardonnay; 2: Pinot gris). The vertical black dashed line separates the Td fermentation alone (left) from the co-inoculated fermentations of Td+QA (right).

**Figure S11.** Kinetics of OH-tyrosol in the two musts extracellular samples (1: Chardonnay; 2: Pinot gris). The vertical black dashed line separates the Td fermentation alone (left) from the co-inoculated fermentations of Td+QA (right).

**Figure S12.** Kinetics of Tryptophol in the two musts extracellular samples (1: Chardonnay; 2: Pinot gris). The vertical black dashed line separates the Td fermentation alone (left) from the co-inoculated fermentations of Td+QA (right).

**Figure S13.** Kinetics of Indole lactic acid in the two musts extracellular samples (1: Chardonnay; 2: Pinot gris). The vertical black dashed line separates the Td fermentation alone (left) from the co-inoculated fermentations of Td+QA (right).

**Figure S14.** Kinetics of Phenyl-lactic acid in the two musts extracellular samples (1: Chardonnay; 2: Pinot gris). The vertical black dashed line separates the Td fermentation alone (left) from the co-inoculated fermentations of Td+QA (right).

**Figure S15.** Kinetics of Indole carboxaldehyde in the two musts extracellular samples (1: Chardonnay; 2: Pinot gris). The vertical black dashed line separates the Td fermentation alone (left) from the co-inoculated fermentations of Td+QA (right).

**Figure S16.** Kinetics of Indole acetic acid in the two musts extracellular samples (1: Chardonnay; 2: Pinot gris). The vertical black dashed line separates the Td fermentation alone (left) from the co-inoculated fermentations of Td+QA (right).

**Figure S17.** Kinetics of Indole acetic acid ethyl ester in the two musts extracellular samples (1: Chardonnay; 2: Pinot gris). The vertical black dashed line separates the Td fermentation alone (left) from the co-inoculated fermentations of Td+QA (right).

**Figure S18.** Kinetics of Indole carbohaldehyde sulfonated in the two musts extracellular samples (1: Chardonnay; 2: Pinot gris). The vertical black dashed line separates the Td fermentation alone (left) from the co-inoculated fermentations of Td+QA (right).

**Figure S19.** Kinetics of 2-aminoacetophenone in the two musts extracellular samples (1: Chardonnay; 2: Pinot gris). The vertical black dashed line separates the Td fermentation alone (left) from the co-inoculated fermentations of Td+QA (right).

**Figure S20.** Kinetics of Kynurenine in the two musts intracellular samples (1: Chardonnay; 2: Pinot gris). For the Td the samples are until the con-inoculation (5 days).

**Figure S21.** Kinetics of Kynurenic acid in the two musts intracellular samples (1: Chardonnay; 2: Pinot gris). For the Td the samples are until the con-inoculation (5 days).

**Figure S22.** Kinetics of Nicotinamide in the two musts intracellular samples (1: Chardonnay; 2: Pinot gris). For the Td the samples are until the con-inoculation (5 days).

**Figure S23.** Kinetics of Tryptophan in the two musts intracellular samples (1: Chardonnay; 2: Pinot gris). For the Td the samples are until the con-inoculation (5 days).

**Figure S24.** Kinetics of Tryptophol in the two musts intracellular samples (1: Chardonnay; 2: Pinot gris). For the Td the samples are until the con-inoculation (5 days).

**Table S1.** Standards

**Table S2.** Descriptive statistics of the extracellular metabolites concentrations in Chardonnay and one way Anova analysis (Separate excel file).

**Table S3.** Descriptive statistics of the extracellular metabolites concentrations in Pinot gris and one way Anova analysis (Separate excel file).

**Table S4.** Descriptive statistics of the intracellular metabolites concentrations in Chardonnay and one way Anova analysis (Separate excel file).

**Table S5.** Descriptive statistics of the intracellular metabolites concentrations in Pinot gris and one way Anova analysis (Separate excel file).

**
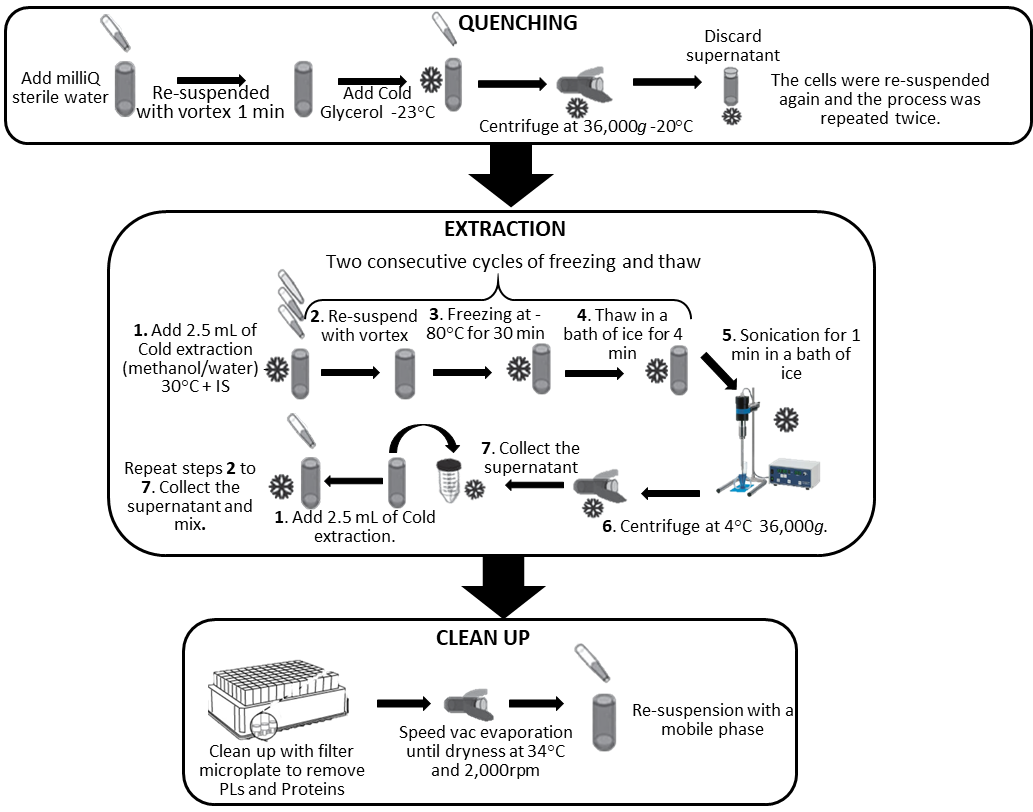
**

**Figure S1.** Scheme of the sample extraction procedure. Based on Villas-Bôas et al.


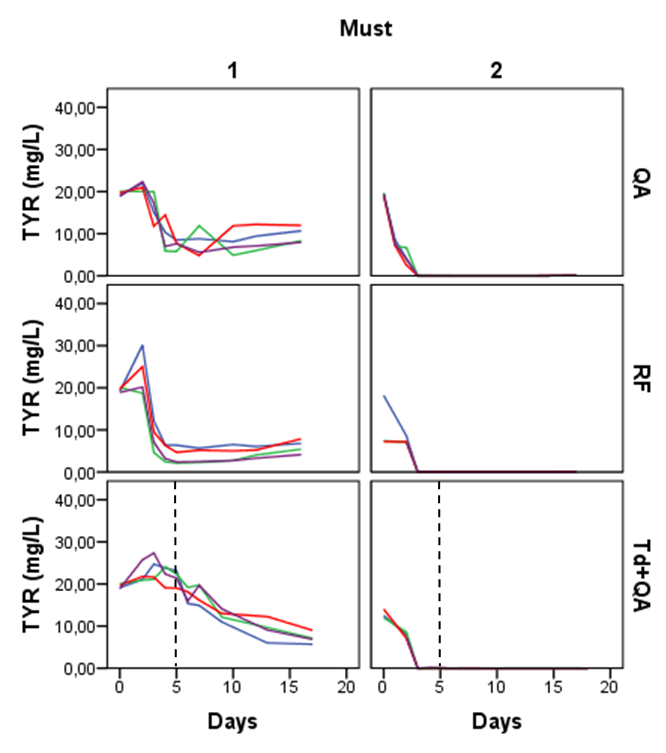


**Figure S2.** Kinetics of Tyrosine in the two musts extracellular samples (1: Chardonnay; 2: Pinot gris). The vertical black dashed line separates the Td fermentation alone (left) from the co-inoculated fermentations of Td+QA (right).


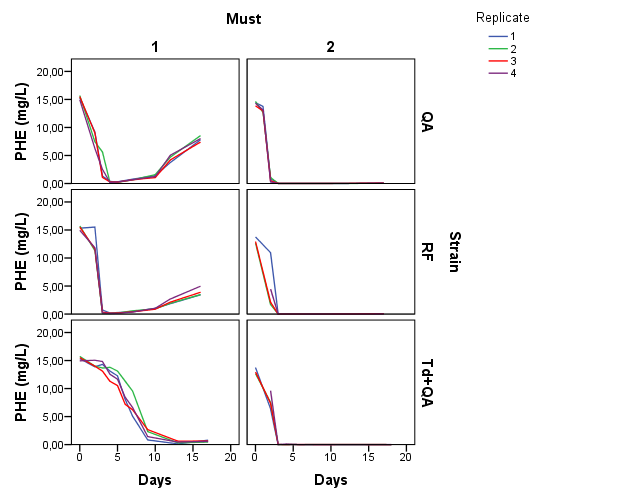


**Figure S3.** Kinetics of Phenylalanine in the two musts extracellular samples (1: Chardonnay; 2: Pinot gris). The vertical black dashed line separates the Td fermentation alone (left) from the co-inoculated fermentations of Td+QA (right).


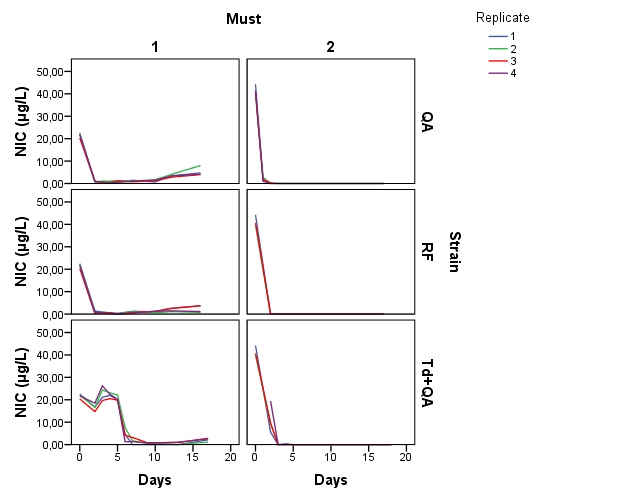


**Figure S4.** Kinetics of Nicotinamide in the two musts extracellular samples (1: Chardonnay; 2: Pinot gris). The vertical black dashed line separates the Td fermentation alone (left) from the co-inoculated fermentations of Td+QA (right).


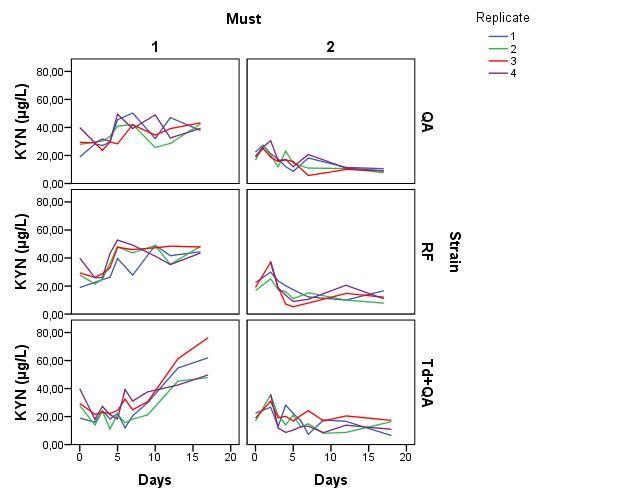


**Figure S5.** Kinetics of Kynurenine in the two musts extracellular samples (1: Chardonnay; 2: Pinot gris). The vertical black dashed line separates the Td fermentation alone (left) from the co-inoculated fermentations of Td+QA (right).


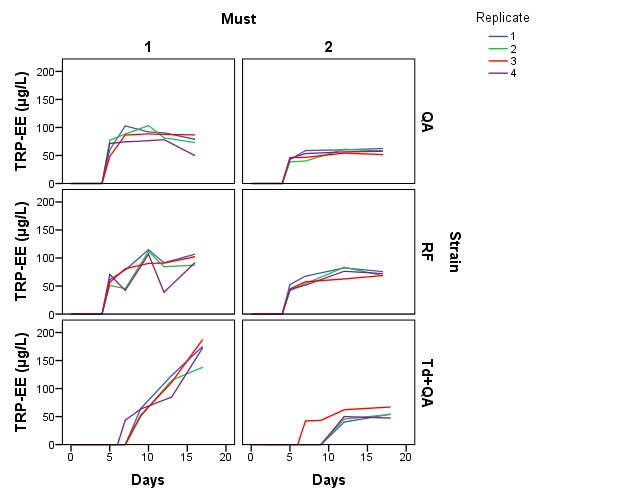


**Figure S6.** Kinetics of Tryptophan ethyl ester in the two musts extracellular samples (1: Chardonnay; 2: Pinot gris). The vertical black dashed line separates the Td fermentation alone (left) from the co-inoculated fermentations of Td+QA (right).


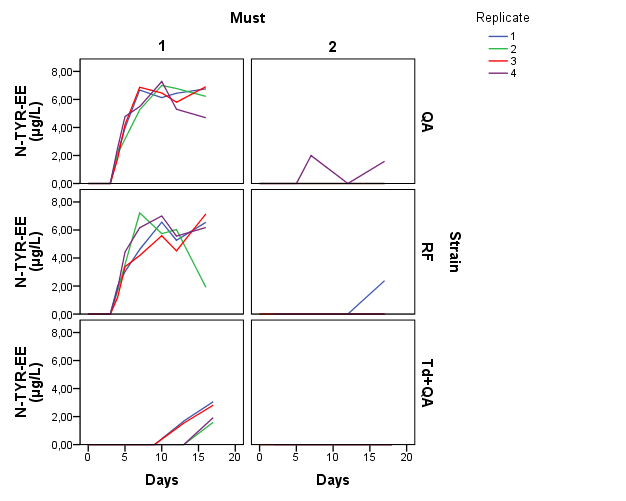


**Figure S7.** Kinetics of N-acetyl-Tyrosine ethyl ester in the two musts extracellular samples (1: Chardonnay; 2: Pinot gris). The vertical black dashed line separates the Td fermentation alone (left) from the co-inoculated fermentations of Td+QA (right).


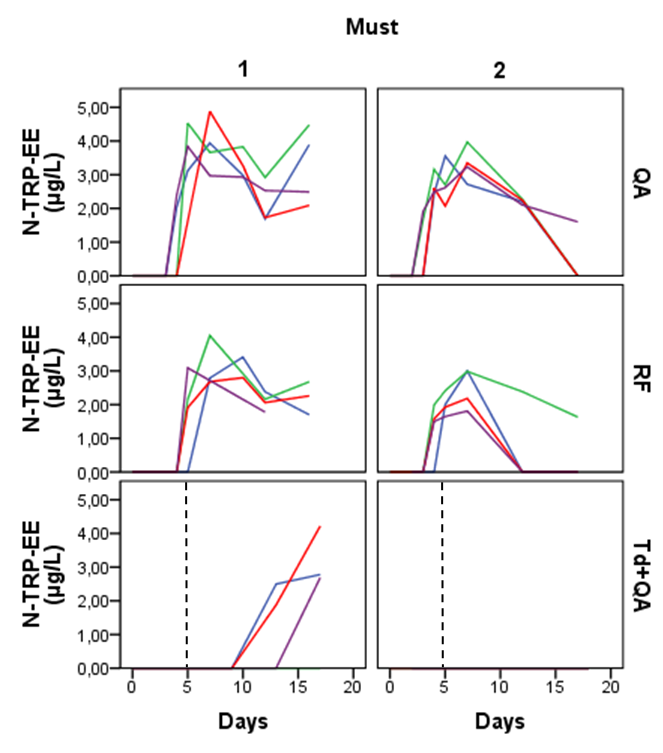


**Figure S8.** Kinetics of N-acetyl-Tryptophan ethyl ester in the two musts extracellular samples (1: Chardonnay; 2: Pinot gris). The vertical black dashed line separates the Td fermentation alone (left) from the co-inoculated fermentations of Td+QA (right).


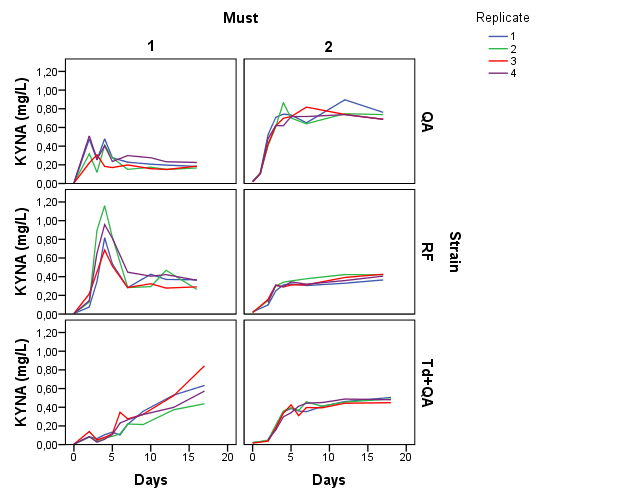


**Figure S9.** Kinetics of Kynureninc acid in the two musts extracellular samples (1: Chardonnay; 2: Pinot gris). The vertical black dashed line separates the Td fermentation alone (left) from the co-inoculated fermentations of Td+QA (right).


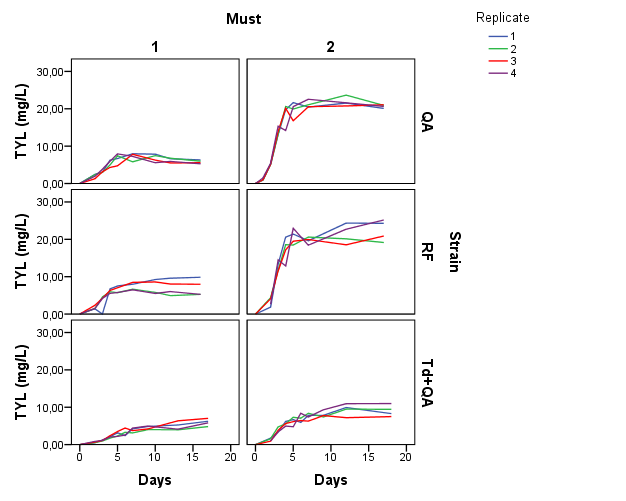


**Figure S10.** Kinetics of tyrosol in the two musts extracellular samples (1: Chardonnay; 2: Pinot gris). The vertical black dashed line separates the Td fermentation alone (left) from the co-inoculated fermentations of Td+QA (right).


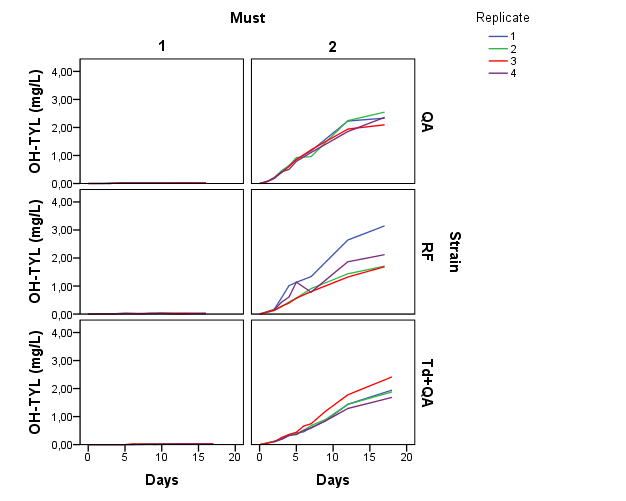


**Figure S11.** Kinetics of OH-tyrosol in the two musts extracellular samples (1: Chardonnay; 2: Pinot gris). The vertical black dashed line separates the Td fermentation alone (left) from the co-inoculated fermentations of Td+QA (right).


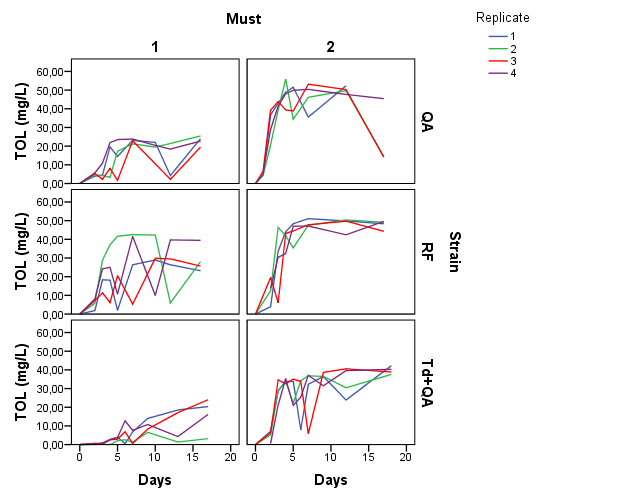


**Figure S12.** Kinetics of Tryptophol in the two musts extracellular samples (1: Chardonnay; 2: Pinot gris). The vertical black dashed line separates the Td fermentation alone (left) from the co-inoculated fermentations of Td+QA (right).


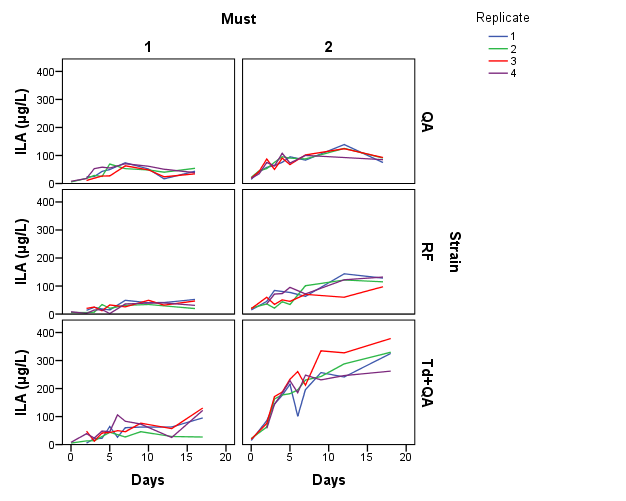


**Figure S13.** Kinetics of Indole lactic acid in the two musts extracellular samples (1: Chardonnay; 2: Pinot gris). The vertical black dashed line separates the Td fermentation alone (left) from the co-inoculated fermentations of Td+QA (right).


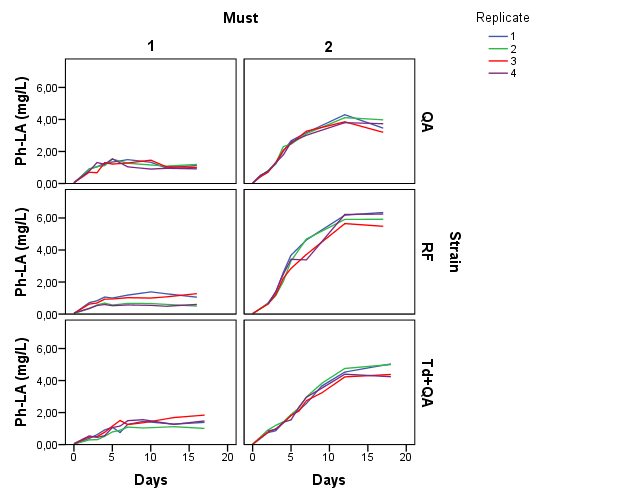


**Figure S14.** Kinetics of Phenyl-lactic acid in the two musts extracellular samples (1: Chardonnay; 2: Pinot gris). The vertical black dashed line separates the Td fermentation alone (left) from the co-inoculated fermentations of Td+QA (right).


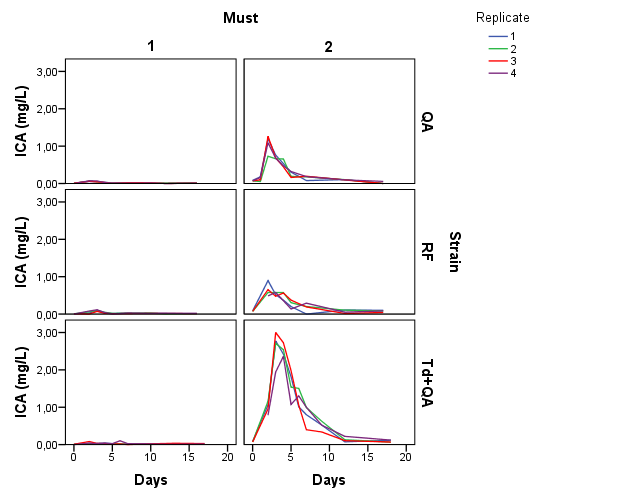


**Figure S15.** Kinetics of Indole carboxaldehyde in the two musts extracellular samples (1: Chardonnay; 2: Pinot gris). The vertical black dashed line separates the Td fermentation alone (left) from the co-inoculated fermentations of Td+QA (right).


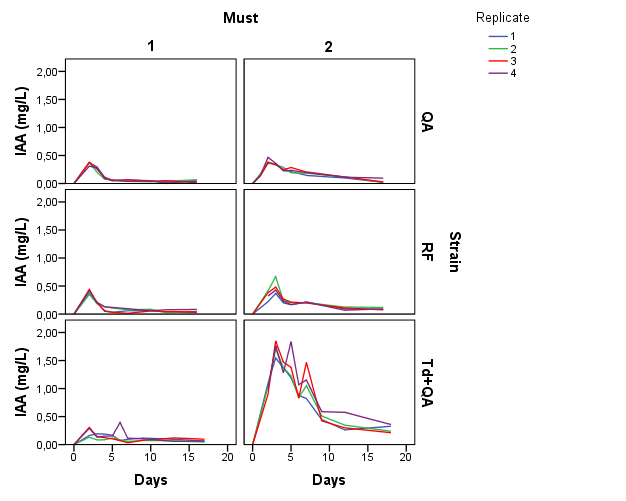


**Figure S16.** Kinetics of Indole acetic acid acid in the two musts extracellular samples (1: Chardonnay; 2: Pinot gris). The vertical black dashed line separates the Td fermentation alone (left) from the co-inoculated fermentations of Td+QA (right).


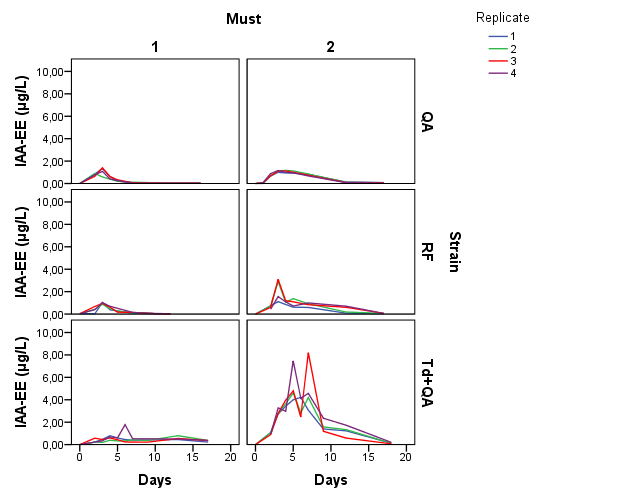


**Figure S17.** Kinetics of Indole acetic acid ethyl ester in the two musts extracellular samples (1: Chardonnay; 2: Pinot gris). The vertical black dashed line separates the Td fermentation alone (left) from the co-inoculated fermentations of Td+QA (right).


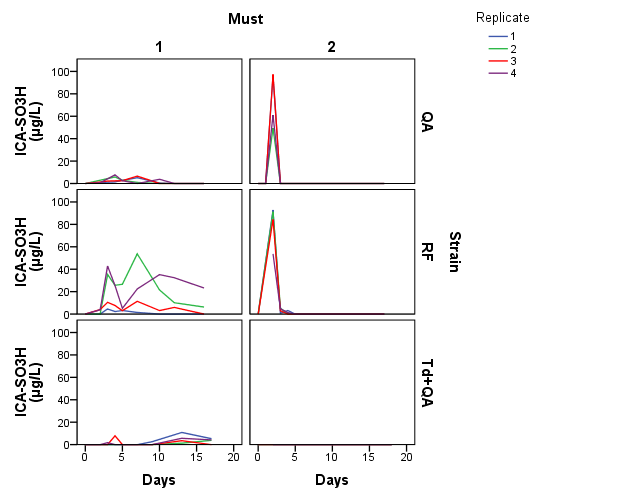


**Figure S18.** Kinetics of Sulfonated Indole carbaxaldehyde in the two musts extracellular samples (1: Chardonnay; 2: Pinot gris). The vertical black dashed line separates the Td fermentation alone (left) from the co-inoculated fermentations of Td+QA (right).


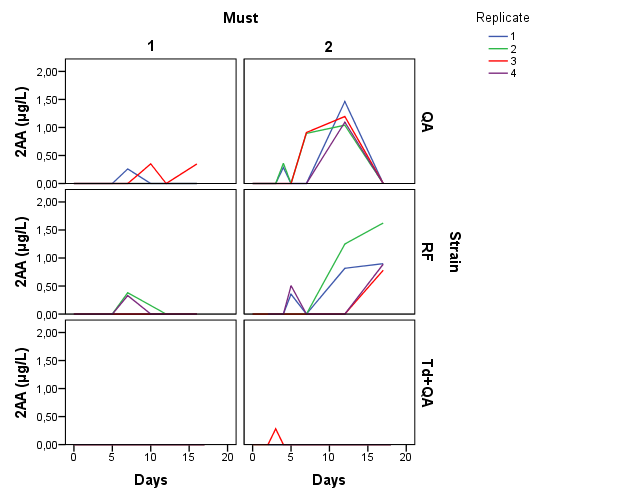


**Figure S19.** Kinetics of 2-aminoacetophenone in the two musts extracellular samples (1: Chardonnay; 2: Pinot gris). The vertical black dashed line separates the Td fermentation alone (left) from the co-inoculated fermentations of Td+QA (right).


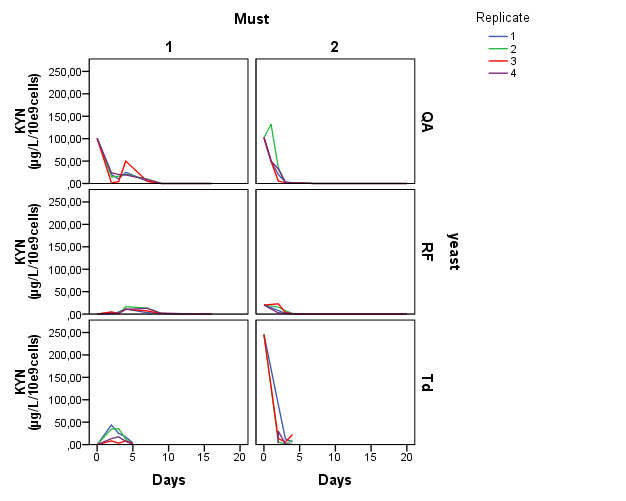


**Figure S20.** Kinetics of Kynurenine in the two musts intracellular samples (1: Chardonnay; 2: Pinot gris). For the Td the samples are until the con-inoculation (5 days).


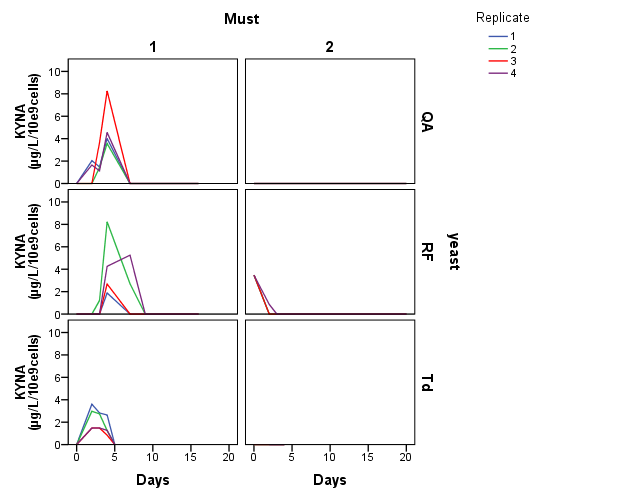


**Figure S21.** Kinetics of Kynurenic acid in the two musts intracellular samples (1: Chardonnay; 2: Pinot gris). For the Td the samples are until the con-inoculation (5 days).


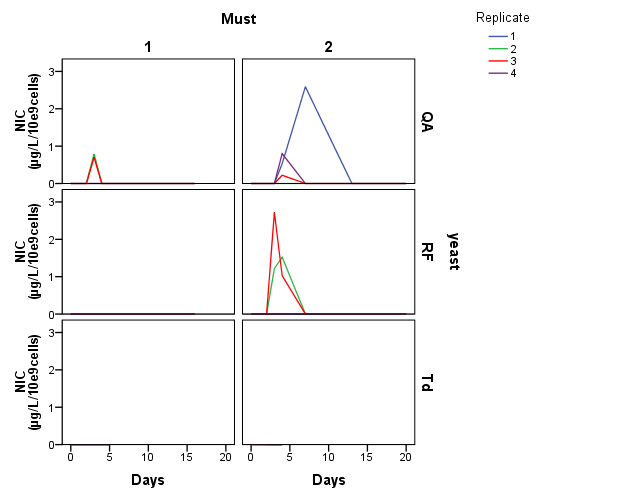


**Figure S22.** Kinetics of Nicotinamide in the two musts intracellular samples (1: Chardonnay; 2: Pinot gris). For the Td the samples are until the con-inoculation (5 days).


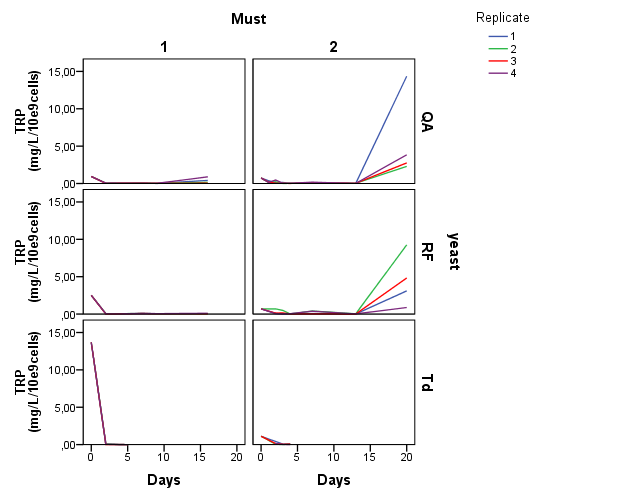


**Figure S23.** Kinetics of Tryptophan in the two musts intracellular samples (1: Chardonnay; 2: Pinot gris). For the Td the samples are until the con-inoculation (5 days).


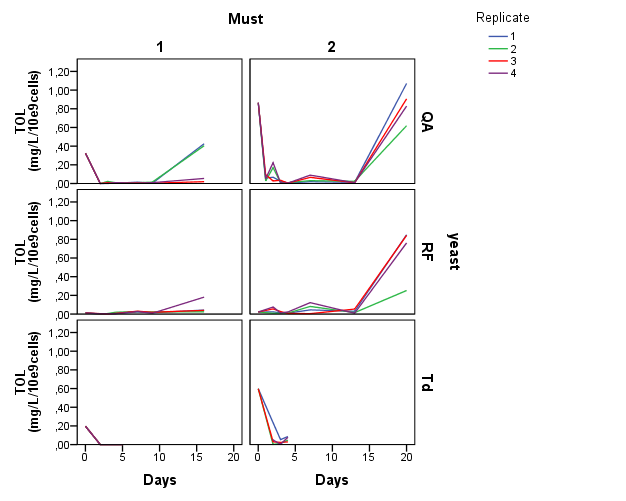


**Figure S24.** Kinetics of Tryptophol in the two musts intracellular samples (1: Chardonnay; 2: Pinot gris). For the Td the samples are until the con-inoculation (5 days).

**Table S1.** Standards

| **Sigma-Aldrich SL Madrid, Spain** | **Sigma-Aldrich, Milan, Italy** | |
| --- | --- | --- |
| tryptamine (TRYPT) | 1-cyclohexene-1-carboxylic acid | phenyl pyruvic acid (Ph-Py) |
| 3-Indole acetic acid (IAA) | 2-amino acetophenone (2AA) | picolinic acid |
| 3-indole butyric acid (IBA) | 2-hydroxy-phenyl acetic acid | shikimic acid |
| 3-indole pyruvic acid (IPy) | 3-ethyl-indole carboxylic acid (E-I Ca) | tryptophan methyl ester (TRP ME) |
| 4-hydroxy-phenyl acetic acid (OH-Ph-AA) | 3-hydroxy kynurenine (OH-KYN) | tyramine (TYRA) |
| 5-hydroxy-L-tryptophan (OH-TRP) | 3-hydroxy-anthranilic acid (OH-ANT) | tyrosine (TYR) |
| 5-Methoxytryptamine (5MOT) | 3-indole acetic acid ethyl ester (IAA-EE) | tyrosine methyl ester (TYRME) |
| 5-Metoxy tryptophan (CH_3_O-TRP) | 3-indole lactic acid (ILA) | formic acid (LC-MS grade) |
| 5-metoxy-3-indole acetic acid (CH_3_O-IAA) | 3-indole propionic acid (IPA) | methanol (LC-MS grade) |
| 5-Metoxytryptophol (5-HTOL | 3-methoxy tyramine |  |
| 6-Hydroxymelatonin (OH-MEL) | 3-methyl-indole (CH_3_-IND) |  |
| DL-kynurenine (KYN) | 3-nitrotyrosine(IS) |  |
| DL-tryptophan methyl ester (TRP-ME) | 3,4-dihydroxy-3-methoxyphenyl propionic acid |  |
| hydroxy indole -3-acetic acid (5H-IAA) | 3(2,4-dihydroxy) phenyl propionic acid |  |
| kynurenic acid (KYNA) | 4-hydroxy-phenyl pyruvic acid (OH-Ph-Py) |  |
| L-Tyrosine (TYR) | 5-methoxy tryptophol |  |
| L-tyrosine methyl ester (TYR-ME) | 6-benzyloxy-6-methoxy indole |  |
| melatonin (MEL) | abscisic acid |  |
| N-acetyl serotonin (N-SER) | anthranilic acid (ANT) |  |
| N-acetyl tryptophan ethyl ester (N-TRP-EE) | dopamine (DOPA) |  |
| N-acetyl-L-tyrosine ethyl ester (N-TYR-EE) | ethyl anthranilate |  |
| phenyl acetic acid (Ph-AA) | indole (IND) |  |
| serotonin (SERO) | indole acetamide |  |
| tryptophan (TRP) | indole acetic acid methyl ester (IAAME) |  |
| tryptophan ethyl ester (TRP-EE) | indole carbinol (I3C) |  |
| tryptophol (TOL) | indole carboxaldehyde (ICA) |  |
| tyrosine ethyl ester (TYR-EE) | indole-2-carboxylic acid |  |
| tyrosol (TYL) | indoxyl sulphate |  |
|  | methyl-indole acetic acid (M-IAA) |  |
|  | nicotinamide (NIC) |  |
|  | nicotinic acid |  |
|  | phenyl alanine (PHE) |  |
|  | phenyl lactic acid (Ph-LA) |  |
